# “Decision-critical” work: a conceptual framework

**DOI:** 10.1186/s12995-016-0115-8

**Published:** 2016-05-06

**Authors:** Xiangning Fan, Charl Els, Kenneth J. Corbet, Sebastian Straube

**Affiliations:** Division of Preventive Medicine, Department of Medicine, Faculty of Medicine and Dentistry, University of Alberta, 5-30F University Terrace, 8303-112 Street, Edmonton, AB T6G 2T4 Canada; Department of Psychiatry, Faculty of Medicine and Dentistry, University of Alberta, Edmonton, AB Canada; Department of Community Health Sciences, Cumming School of Medicine, University of Calgary, Calgary, AB Canada

**Keywords:** Decision-critical work, Safety-sensitive work, Fitness to work, Accidents, Occupational safety

## Abstract

“Safety-sensitive” workers, also termed “safety-critical” workers, have been subject to fitness to work assessments due to concerns that a performance error may result in worker injury, injury to coworkers or the general public, and/or disruption of equipment, production or the environment. However, there exists an additional category of “decision-critical” workers, distinct from “safety-sensitive” workers, in whom impairment may impact workplace performance, relationships, attendance, reliability and quality. Adverse consequences in these latter areas may not be immediately apparent, but a potential “orbit of harm” nevertheless exists. Workplace consequences arising from impairment in “decision-critical” workers differ from those in “safety-sensitive” personnel. Despite their importance in the occupational context, “decision-critical” workers have not previously been differentiated from other workers in the published literature, and we now outline an approach to fitness to work assessment in this group.

## Background

A “safety-critical” task has previously been defined to mean “one where certain forms of personal impairment can put other people at risk” [1]. The terms “safety-sensitive” and “safety-critical” have been used in a number of recent occupational health guidance documents [2, 3], outlining the importance of this group of workers. The concept of risk in this operational definition has been further refined in case law to include risks both to the worker as well as to others arising out of performance error due to physical or mental conditions, with consideration of the nature, magnitude and immediacy of the risks. The broader category of “safety-sensitive” work can be thought of as encompassing work in which one or more “safety-critical” tasks are or may be performed, and where possible consequences include death or serious injury of a worker or a member of the general public, or, alternately, damage to or serious disruption of equipment, production or the environment. Of note, opportunities to mitigate harms arising from worker impairment in a “safety-sensitive” role may be limited, as adverse consequences typically occur within a short period of time following a performance error. In this review, we will build on this framework of “safety-sensitive” work, and develop a distinct concept of “decision-critical” work.

## Review

The distinction between “safety-sensitive” and “non safety-sensitive” work has been used to justify 1. skills certification requirements for workers on a periodic basis, 2. workplace drug and alcohol policies and testing programs, and 3. more stringent and frequent medical assessments, especially in regulated industries (e.g. transportation). Occupational health professionals evaluating “safety-sensitive” workers are required to document a higher threshold of certainty that no medically-based impairment exists or may be reasonably foreseen to exist. However, there exists considerable ambiguity in the exact meaning of “safety-sensitive” work across industry and occupational categories, as well as among medical practitioners. Outside of administrative definitions in regulated industries, there is no clear consensus on the exact boundary between “safety-sensitive” versus “non safety-sensitive” work. Clarity in this regard would enable a consistent, fair and transparent approach to the assessment of “safety-sensitive” workers, and benefit occupational health professionals, workers, employers and public interest.

Despite the importance of the “safety-sensitive” concept, the distinction between “safety sensitive” and “non safety-sensitive” workers is far from the sole delineation of importance when evaluating workers for fitness to work. Given the explicit boundaries of “safety sensitive” work as outlined above, we propose that there exists a second category of “decision-critical” workers, whose continued occupational performance depends on the ability to consistently exercise judgment and insight, but who do not precisely fall under the “safety sensitive” category. Examples of such “decision-critical” workers include corporate executives, schoolteachers, lawyers, information technology workers, and some health professionals, among others. For “decision-critical” workers, adverse workplace consequences may arise from a state of low-grade impairment as well as from a single event or error, with serious consequences that may not be immediately apparent. “Decision-critical” work may affect workers’ wellbeing and livelihoods, and impact employer oversight and stewardship of products and services, but without the same direct and near-term adverse effects as “safety-sensitive” work. Despite the less dramatic workplace consequences, impairment in “decision-critical” workers (particularly of the neurocognitive variety) can still pose workplace difficulty with coworkers, supervisors and clients in the domains of attendance, performance, and workplace relationships, and result in financial, legal, reputational or psychological harm, and/or corporate liability. We propose that one definition of “decision-critical” workers is workers whose continued occupational performance depends on the ability to consistently exercise judgment and insight in the workplace, but who would not be considered “safety-sensitive” workers. The case vignettes in the Appendix of this review provide examples of workers who may be considered “decision-critical” and detail how medical conditions in “decision-critical” workers can impact occupational function.

Of note, it is generally accepted that a “safety-sensitive” worker’s right to self-determination can be justifiably limited in some circumstances for employers’ and the public’s interest. However, it is not clear to what extent the same applies to “decision-critical” workers when lesser degrees or different kinds of risk exist, such as when the potential harm is property damage, digital information loss, proprietary breaches, legal liability, delayed completion of time-sensitive job tasks, or economic loss. Indeed, while the language of “safety-sensitive” work is inherently connected to the concept of workplace risk, it is not clear whether the discussion for “decision-critical” workers should always continue to be framed in terms of risk when the potential adverse consequences are less overt and not immediately injurious.

Guidance for occupational health professionals on the topic of fitness to work in both “safety-sensitive” and our proposed “decision-critical” workers has been inconsistent to date. Serra and colleagues [4] have previously reported that, of published guidelines on the assessment of fitness to work, approximately 25 % did not describe the decision-making process by which occupational health professionals should evaluate workers at all, and an additional 25 % indicated that the physician “forms an opinion, or arrives at a clinical judgment,” without providing further detail. Of 39 articles on the assessment of fitness to work identified, 34 discussed “health and safety risk,” and 31 outlined “determination of capacity.” However, of the latter, only 11 addressed the worker’s psychological capacity and it is not clear how many guidelines separately considered neurocognitive ability as it applies to decision-making processes.

We propose that the process of assessing fitness to work in “safety-sensitive” and “decision-critical” workers include the explicit identification of important neurocognitive domains from essential job tasks, in the same way that physical demands are separately considered within a physical job demands analysis (e.g. for functional capacity assessment). Indeed, for some “safety-sensitive” and “decision-critical” functions (e.g. driving [5–9]), this has already been attempted in generally applicable medical guidelines. In the workplace, existing cognitive and behavioural job demands analyses (e.g. utilized by the City of Toronto in Toronto, Ontario, Canada [10]) necessitate clear definitions and field-testing to ensure validity [11] but hold promise for standardizing the translation of workplace demands into the health domain (and vice versa). For both “safety sensitive” and “decision-critical” workers, the medical assessment must be relevant, valid, and reliable, comparing the worker’s capabilities to the demands of the job. Where neuropsychiatric testing is available, more detailed information on neurocognitive capability can be assessed than by clinical interview alone, although clinical assessment is likely to be the mainstay of assessment for most workers due to practical considerations.

**Fig. 1:**
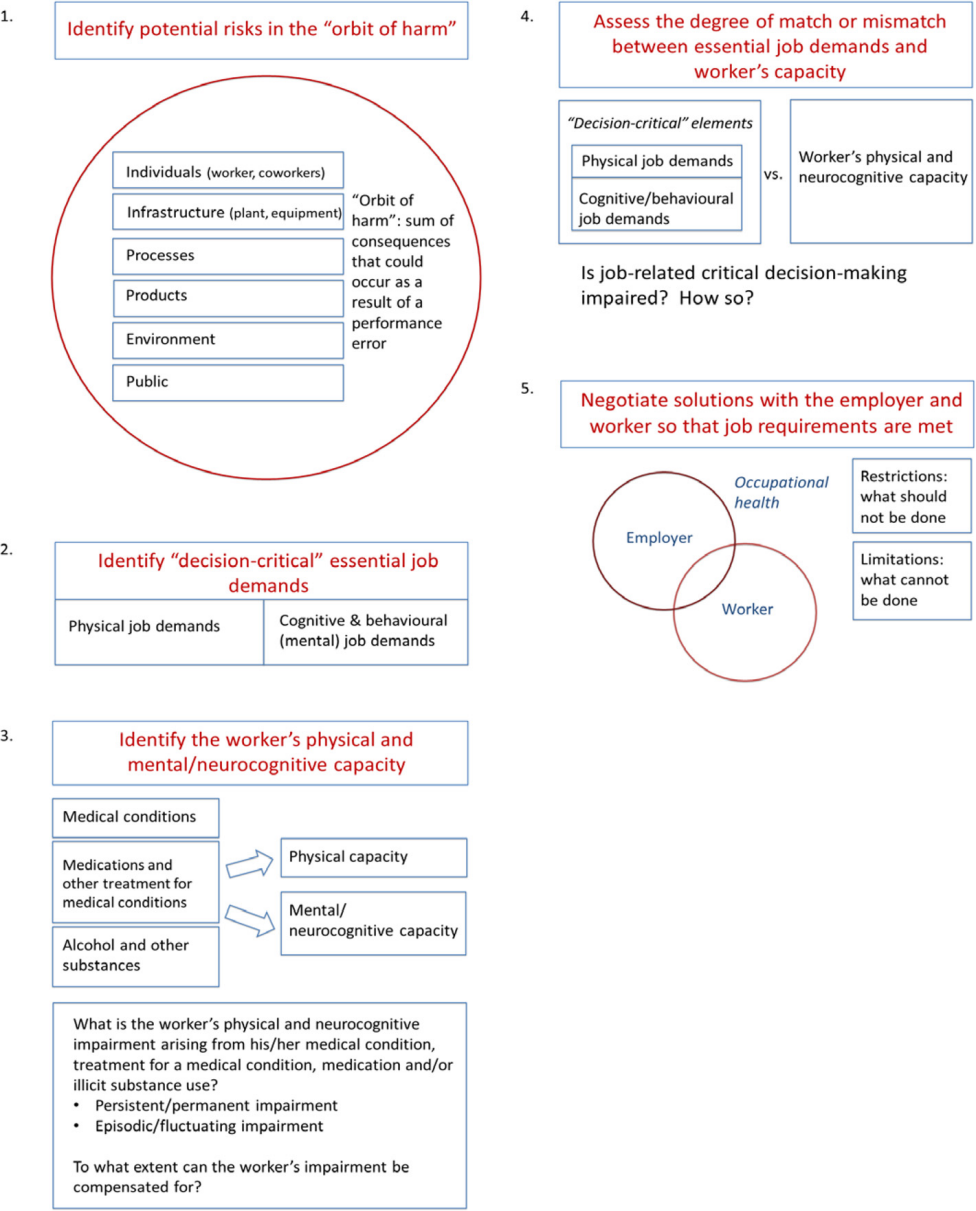
Proposed stepwise approach for assessing “decision-critical” workers. Details of individual steps are outlined in the article text

Both episodic and persistent phenomena from medical conditions should be identified in the fitness to work assessment of “safety-sensitive” and “decision-critical” workers. Pertinent conditions may include mental health conditions, personality disorders, and somatic diseases (such as endocrine or cerebrovascular conditions) which may interfere with one or more neurocognitive domains. The worker should be asked about use of medications (e.g. opioids [2]) or illicit substances [12] generally held to preclude “safety-sensitive” work due to impairment of alertness, cognition or judgment. In the context of his/her medical condition, the ability of the worker to function, with or without job accommodation, should then be considered. It has been argued for psychiatric illness [13], and is also true of medical conditions in general, that functional impairment and decision-making capacity should be considered separately from the condition itself. We therefore recommend the following step-wise approach (see Fig. 1):

1. Identify risks in the “orbit of harm” including potentially affected individuals, infrastructure, processes, products, the environment or the public.
2. Identify the “decision-critical” elements of the job and isolate physical and cognitive/behavioural essential job demands.
3. Assess the worker’s physical and neurocognitive capacity by clinical examination or appropriate neuropsychological testing. Capacity can be adversely impacted by a medical condition, treatment for a medical condition, medication side effects, or use of alcohol and/or illicit substances. Include assessment for both persistent/permanent impairment and episodic/fluctuating impairment arising from the above factors.
4. Analyze the degree of match or mismatch between “decision-critical” essential job demands and medical/neurocognitive capacity (impairments). Is job-related critical decision making impaired and, if so, how severely?
5. Negotiate solutions with the employer and worker so that job requirements are met. This may include a formulation of restrictions and limitations [14].

One implication of the distinction between “safety-sensitive” and “decision-critical” workers may be that some workers who are currently classified as “safety-sensitive” should be reclassified as “decision-critical” workers. Another implication of this new categorization of workers is that additional fitness to work evaluation frameworks may be required to assist occupational health professionals in evaluating “decision-critical” workers, as current guidance has been written for “safety-sensitive” or “safety-critical” workers [2, 3], or workers at large. Finally, it is unclear whether certain programs and policies applicable to “safety-sensitive” workers (e.g. workplace drug and alcohol policies and testing programs) can be justified for “decision-critical” workers as well.

## Conclusions

To our knowledge, the classification of “decision-critical” workers as a distinct group from “safety-sensitive” workers has not been described previously in the published literature, but this separation provides clarity and utility for occupational health practitioners. Firstly, such a category would allow a narrower and conceptually clearer definition of “safety-sensitive work.” Secondly, our description of “decision-critical” work identifies a group of workers whose fitness for work has important occupational and societal implications, and who have not so far been the focus of occupational health professionals. An existing occupational health practice of performing medical assessments of “safety-sensitive” workers can be applied to a broader range of workers in “decision-critical” occupations. We hope the methodology we outline will add validity and reliability to the fitness evaluation of workers in occupations where decision-making is an essential job requirement (e.g. physicians [15]). An advantage of this approach is to convert some questions of putative risk to those of capacity, as these terms are outlined in the popular reference by the American Medical Association [14], and assist treating clinicians in formulating specific, actionable recommendations about fitness to work. The approach we outline is empirical and clinical, as research on developing well-validated measures of cognitive and behavioural job demands, and cognitive ability is ongoing. Judgment and insight are difficult to formally assess by generalist physicians, and poor insight and judgment may exist in the absence of an underlying medical condition. Formal validation of the utility of differentiating between “safety-sensitive” and “decision-critical” workers is required through future research.

## Appendix

### Case vignettes

#### Case vignette 1

A Lawyer in a large family legal practice has been observed consuming alcohol over his lunch hour and, at times, in his office throughout the afternoon. On several occasions he was inaccessible to senior partners, clients, and family members. His appearance in the workplace and regular absences commonly resulted in frequently missed deadlines, miscommunication with clients and partners, and inaccuracies in legal documents. After several meetings with the managing partners regarding his absenteeism and the impact of alcohol use on his work, he was terminated from his position.

**Discussion:** Professional workers [16] (e.g. lawyers [17, 18] and physicians [19, 20] and workers in higher occupational grades [21], many of whom would be considered “decision-critical” workers, are at risk of substance use disorders, and may encounter special barriers to proper assessment and treatment. An untreated Alcohol Use Disorder may adversely impact an individual’s cognitive processes, behaviour, and presentation. This may result in substantial disruption in professional occupational activities, to the detriment of clients, organizations and institutions. Social stigma may deter highly functioning “decision-critical” workers from seeking help for problematic alcohol consumption or substance use, leading to a delay in diagnosis and treatment during which the “decision-critical” worker may continue in a workplace role with substantial responsibilities.

#### Case vignette 2

A Store Manager working in a community mall presented with a 2-year history of episodes of forgetfulness. She additionally reported sleep disturbance, and decreased energy. Her symptoms made it difficult for her to process orders and inventory, oversee staff, and, on one occasion, secure the store at the time the fire alarm was sounded. Part of her duties included being the person in charge of responses to emergencies in the store. She was diagnosed with Alzheimer’s disease after a neurological assessment.

**Discussion:** Workers with cognitive disorders (e.g. dementia) may present for a fitness to work assessment due to concerns about consistency, judgment, cognitive capacity, and predictability of mental functioning. Some occupational groups (e.g. lawyers, teachers and counsellors) are highly “decision-critical” in having significant cognitive and behavioural job requirements, and workplace performance concerns may be a presenting feature of cognitive disorders in such workers. The physician who is asked to assess a worker who holds a “decision-critical” post should elucidate the specific occupational requirements and attempt to determine whether the worker’s functional ability to meet the essential job demands is impaired. Of note, it may be that a “decision-critical” worker is required to exercise insight and judgment only during exceptional workplace events, not on an everyday basis.

#### Case vignette 3

A Team Leader in a high-profile advertising firm with a history of anxiety reported excessive worries about an upcoming internal company merger. He expressed that his worries about daily activities (i.e., chores, self care, finances) were out of control and were exacerbated by the thought of incorporating additional employees under his oversight. He was often preoccupied with extraneous tasks and thoughts when faced with having to restructure his department. His worries often led to physical symptoms including feelings of panic, upset stomach, shortness of breath, and increased psychomotor agitation.

**Discussion:** Individuals with Generalized Anxiety Disorder demonstrate impaired judgment, inattention, and fatigue, which may result in substantial impairment in occupational duties. Additionally, somatization may be a component of the presentation, and lead to an extensive work-up for an underlying medical condition (e.g. a work-up for cardiac causes of chest pain), which can raise concerns about fitness to work in both “safety-sensitive” and “decision-critical” roles.

#### Case vignette 4

A Surgeon was noted to behave erratically in his interactions with patients. Additionally, he had had a number of interactions with coworkers that were perceived as disrespectful, raising concerns as to his ability to continue to function safely in the hospital. A complaint was made to his regulatory college. He was referred for an assessment of his fitness to practice, and diagnosed with a major mood disorder, which had impacted his cognitive capacity. After treatment, he was able to successfully return to the operating theatre, albeit under supervision. The prescribed antidepressants had the potential to result in tremor and sedation, necessitating ongoing assessment of his physical and mental status.

**Discussion:** Among health care workers, some (e.g. surgeons and anaesthesiologists in the operating theatre) may be considered “safety-sensitive”, while others (e.g. with largely administrative or academic roles) may fall into the “decision-critical” category. The distinction can be difficult to make, but in cases where the likelihood of direct patient harm as a result of performance error is high, it may be reasonable to consider such health care workers to be “safety-sensitive” workers. The exact stratification may require a detailed review of job tasks and the working environment.
